# Wireless Monitoring of Changes in Crew Relations during Long-Duration Mission Simulation

**DOI:** 10.1371/journal.pone.0134814

**Published:** 2015-08-07

**Authors:** Bernd Johannes, Alexej S. Sitev, Alla G. Vinokhodova, Vyacheslav P. Salnitski, Eduard G. Savchenko, Anna E. Artyukhova, Yuri A. Bubeev, Boris V. Morukov, Carole Tafforin, Mathias Basner, David F. Dinges, Jörn Rittweger

**Affiliations:** 1 Division of Space Physiology, Institute of Aerospace Medicine, German Aerospace Center (DLR), Cologne, Germany; 2 Division of Psychophysiology and Neurophysiology of operator’s activity, State Research Center of Russian Federation, Institute for Biomedical Problems RAS, Moscow, Russia; 3 State Research Center of Russian Federation, Institute for Biomedical Problems RAS, Moscow, Russia; 4 Research and Study Group in Human and Space Ethology, Ethospace, Toulouse, France; 5 Department of Psychiatry, University of Pennsylvania Perelman School of Medicine, Philadelphia, Pennsylvania, United States of America; Institutes for Behavior Resources and Johns Hopkins University School of Medicine, UNITED STATES

## Abstract

Group structure and cohesion along with their changes over time play an important role in the success of missions where crew members spend prolonged periods of time under conditions of isolation and confinement. Therefore, an objective system for unobtrusive monitoring of crew cohesion and possible individual stress reactions is of high interest. For this purpose, an experimental wireless group structure (WLGS) monitoring system integrated into a mobile psychophysiological system was developed. In the presented study the WLGS module was evaluated separately in six male subjects (27–38 years old) participating in a 520-day simulated mission to Mars. Two days per week, each crew member wore a small sensor that registered the presence and distance of the sensors either worn by the other subjects or strategically placed throughout the isolation facility. The registration between two sensors was on average 91.0% in accordance. A correspondence of 95.7% with the survey video on day 475 confirmed external reliability. An integrated score of the “crew relation time index” was calculated and analyzed over time. Correlation analyses of a sociometric questionnaire (r = .35-.55, p< .05) and an ethological group approach (r = .45-.66, p < 05) provided initial evidence of the method's validity as a measure of cohesion when taking behavioral and activity patterns into account (e.g. only including activity phases in the afternoon). This confirms our assumption that the registered amount of time spent together during free time is associated with the intensity of personal relationships.

## Introduction

Team work has become a central issue in a variety of professions, e.g. in business [1, 2], for fire fighters [3], in the military [4–6], during space flight [7], overwintering in Antarctica [8, 9], in science [10], sports [11, 12]), and “countless other domains” [13]. The success of complex performance requires the functioning of entire teams rather than of individuals. During manned space flight small crews are exposed to long periods of autonomy, isolation, and confinement [14–16]. Therefore, it is of paramount importance to ensure reliable crew performance and individual well-being for the success of long-duration space missions. Crew cohesion and its dynamics play a central role when coping with extreme physical, social and psychological conditions, with reduced communication, periods of high workload and periods of monotony and boredom. Friendly and successful operational interactions of all crew members are known to be very important under those conditions [17, 18]. However, often “interpersonal problems among crew members of isolated and confined groups are inevitable” [19].

Crew cohesion can be separated into social cohesion, related to the personal relationships, and task cohesion, related to mission goals and success [20]. Meta-analyses [21–23] have shown that cohesion enhances performance in small teams and extremely negative attitudes and interpersonal conflicts are bound to erode team cohesion. On the other hand, team cohesion is not the only relevant factor for mission success, as moderate amounts of conflict can also help to enhance team performance when team members correct each other’s perceptions, offer alternatives, or argue about how to solve a problem. Interpersonal tension and group cohesion have also been demonstrated in space analogue environments [8, 24], and are crucial for mission success during low Earth orbit (LEO) space missions. However, the relationship between crew cohesion and crew performance is not as simple as discussed by Dyaram & Kamalanabhan [25].

Manzey [26] stressed the necessity for future research and development of methods to monitor “interpersonal relationships and crew cohesion”, including the time-course of these effects. After year 2000 the first approaches to analyze the commercial efficiency of work teams by means of badges were described [27–30]. This measurement, based on infrared technology, allowed the duration of face-to-face situations to be registered. Recently a study using this technology was published by Watanabe, Ishibashi & Yano [31] indicating that group performance is interrelated with the frequency of face-to-face interpersonal interactions. Another approach to assess social relationships is based on speech registrations by mobile systems [32–34]. One of the latest developments is ActiWatch that registers proximity of other watches through BlueTooth technology (http://www.actigraphcorp.com/products/wactisleep-bt-monitor/).

During the same period statistical methods for analyzing social relationships showed an enormous development, specifically the “statnet” package for the data analysis system R. (for details see http://www.r-project.org). The statnet core team (David R. Hunter, Carter T. Butts, Steven M. Goodreau, Pavel N. Krivitsky, Skye Bender-deMoll, Martina Morris) published theoretical foundations, analyses of applied data as well as software descriptions and tutorials [35–40]. Snijder and his group worked on stochastic models for network dynamics [41–42]. However, all of these methods are based on binary data (“presence” vs. “no presence”) [43]. Krivitsky and others of the statnet core team therefore began to develop analytical methods for “weighted edges” or “valued ties”–at first for counts of choices [44–46] including a helpful tutorial for their package [47].

For the development of crew support systems for autonomous crews on any kind of long-duration mission [48] a system is needed that can provide objective feedback on crew cohesion to the crew itself. The main aim of such monitoring would be the avoidance of isolation of single crew members (isolation in isolation). Another important aspect would be any reliable prediction of crew cohesion changes at least for the near future. Therefore, an experimental system for objectively monitoring crew cohesion and its time-dependent changes was developed. The sensor hardware represents a module of a mobile polygraph system, which has already been applied in space applications (HealthLab, Koralewski Industrie Elektronik, Hambühren, Germany). This new module was tested for data reliability and validity including the actigraphy measurement, prior to its system-synchronized combination with the other (physiological, voice, and environmental) measurements [49–51] for future studies. The aim was to register the presence of any other sensor in a certain distance range with a sample rate, sufficient for comparisons to physiological reactions. To the author’s knowledge this has never been published and might enhance the possibilities in group research. These feasibility, reliability, and validity tests were performed in the Mars520 project, simulating a manned space flight to Mars and back (in Moscow, IBMP [52–53]; described briefly below).

### Measure of crew relations

The methodology of the Wireless Group Structure tool (WLGS-tool) is based on Moreno’s sociogram [54], a graph that describes the inter-individual relationships among group members. Moreno’s group members had to select and to reject one (seldom two) other crewmember(s) for various future activities, (e.g., a new mission, certain professional task, joint holidays or other activities in their spare time). From this, choices indicators were derived to describe the position of single members within the group, and the overall group cohesion. This method defines the number of selections and rejections of other crew members (Eqs 1 and 2) for a certain task as the intensity of the relationship between these two members.

$$S_{A}=\frac{s_{A}}{N-1}$$ (1)

Selection status of subject A (S_A_) = number of selections s_A_ / crew size (N) –1

$$R_{A}=\frac{r_{A}}{N-1}$$ (2)

Rejection status of subject A R_A_ = number of rejections / crew size –1

A crew cohesion index (Eq 3) was developed, integrating the individual choices.

$$C=\frac{S_{AB}}{N(N-1)/2}$$ (3)

Crew cohesion (C) = number of two-sided selections between crew members (s_AB_) in relation to the number of possible couples N(N-1) /2

In Moreno’s approach the crew cohesion is assumed to be highest when two crew members mutually select each other. This index provides comparable information about relation intensity between subjects within one crew and is comparable between different crews, yielding values between 0 and 1. Moreno discussed the use of time measures as a more detailed measure of relationships than categorical choices [55], however the time measure he had in mind was the subjectively estimated time a subject would like to spend with another one. In analogy to Moreno’s original equations, to the presented study registered the real time spent together (Eq 4) as an indicator of relation intensity.

$$T_{AB}=\frac{t_{AB}}{T}$$ (4)

T_AB—_relative time of crew members A and B spent together;

t_AB_—absolute time of crew members A and B spent together;

T—total time of registrations

The pairwise time spent together was adjusted according to the whole time of registration among all crewmembers, providing an integrated measure of crew-time relations (Eq 5). The index is dimension less and provides a measure of time spent together in relation to the maximum possible time. The underlying assumption is that sharing time reflects inter-personal attraction, and that this can be exploited to reflect the intensity of intra-group *T*_*AB*_ = *t*_*AB*_ / T relationships. Notably, this is only indication of the “selection” case (Eq 1), and the “rejection” case (Eq 2) is not directly amenable to such analysis.

$$CRTI=\frac{\sum_{i=1}^{N-1}t_{XY}}{T^{*}N^{*}(N-1)/2}$$ (5)

CRTI—Crew Relation Time Index

∑t_XY_ Sum of time of any two of N crew members spent together;

T*N*(N-1) / 2 total time multiplied by the number of interrelations (for 6 crew members = T*15)

Within this context, it is important to discern between working time and spare time confirming the necessity of different concepts for task cohesion and social cohesion [20]. Even though the initial aim was to construct a general indicator of cohesion it will be demonstrated in this study that the CRTI is clearly related to other cohesion measures only during spare time.

Moreover, by installing sensors at certain locations within the habitat, it is possible to characterize the geography of group cohesion. For example, sensors installed in common rooms or private rooms will indicate social interactions in relation to housekeeping, cooking in the kitchen, the main social hall, inside the physical training facility, etc.

The aim of this study was to test the reliability and validity of this objective monitoring approach for group structure and its changes in a space analogue. If reliability and validity can be confirmed, one could use automated analyses of the relationship data and combine this kind of data with other physiological measurements for monitoring purposes. The time measurements were validated with social psychological, ethological, and psychosocial methods. A classical sociometric questionnaire, video analyses and actigraphy data were used for validation. Finally, it was attempted to develop practical and relevant methods of visualization and analysis of group structure changes based on the obtained objective continuous data, including a first approach for predicting the crew relation development for the next few days.

## Methods

### Study design

This study was part of the Mars500-project, a 520-day simulated return mission to Mars performed in the terrestrial experimental complex (NEK) at the Institute of Biomedical Problems (IBMP) in Moscow, Russia (2009–2011) and was supported by the European Space Agency (ESA) and the German aerospace Center (DLR). A 105-day pilot study preceded the 520-day study. Six international crew members participated in each study. A detailed report on the design of the Mars500-project has been published by IBMP and ESA [53–54]. The data reported in this manuscript were gathered in the 520-day study.

### Participants

Six young and healthy men (3 Russians, 2 West-Europeans, 1 Chinese) participated in the 520-day study of the Mars500-project. Crewmembers were randomly assigned to the letters A-F. This coding followed the same scheme used by Basner et al. [56, 57] in their Mars500-experiment (actigraphy) and thus allows the reader to compare subjects across publications.

### Ethical approval

Ethical approval was obtained from the local institutional review boards of the Academy of Science, Moscow, Russia, and ESA. All participants gave their written informed consent prior to the start of the study.

### Protocol

For the WLGS experiment one training and instruction session were required prior to the start of the study for the one participant who was responsible for the conduction of the experiment inside the isolation chamber. The testing equipment was deployed twice weekly (Tuesday and Friday) with all six participants wearing sensors and six sensors placed in the main habitat rooms.

### Equipment

The sensors hardware was based on a prototype of psychophysiological measurement satellites under the control of a master developed and available by the KORALEWSKI Industrie Elektronik (Hambuehren, Germany) and funded by DLR. The software was developed and available by SpaceBit GmbH (Eberswalde, Germany).

**Fig 1:**
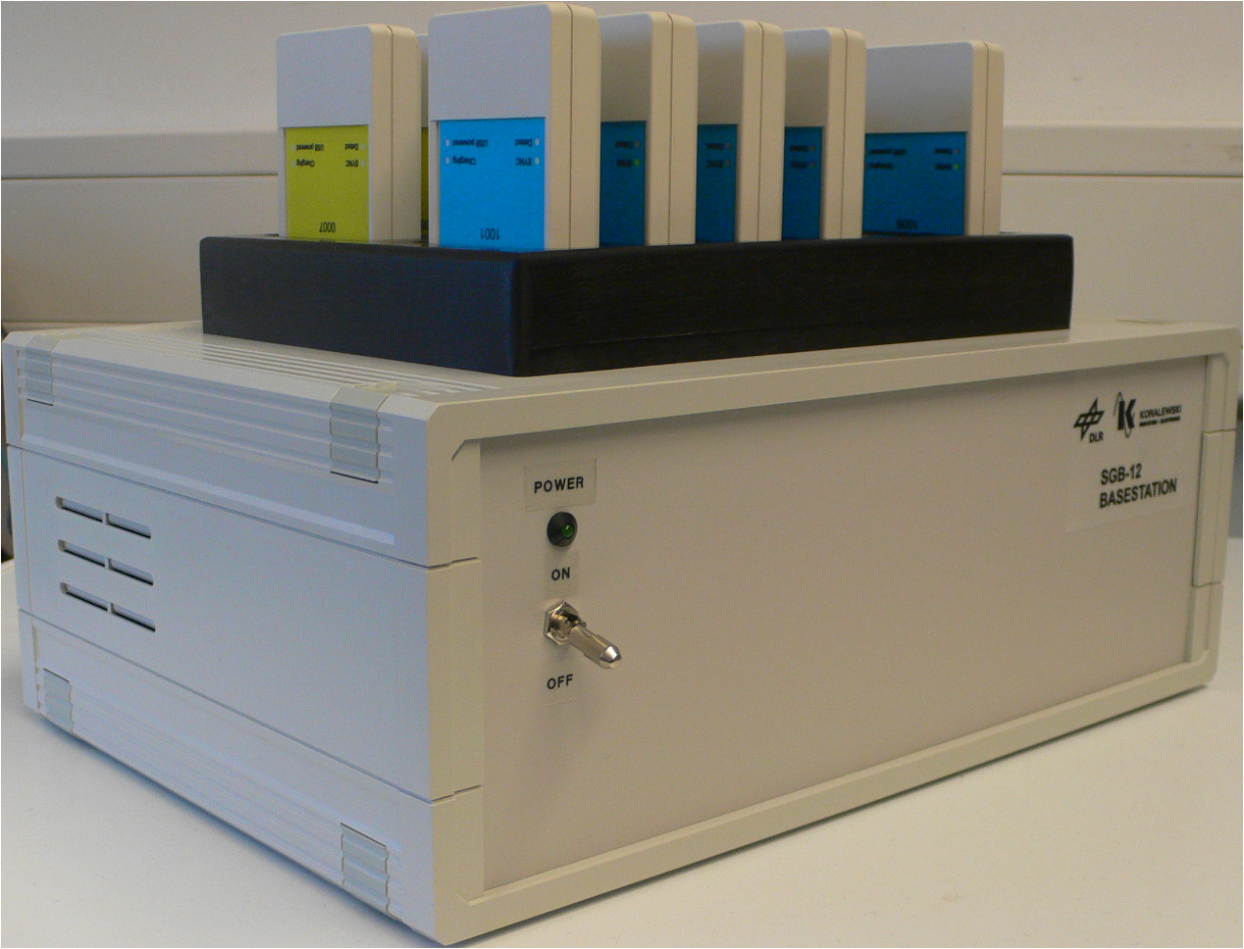
WLGS hardware. Wireless Group Structure (WLGS) system, base station with 12 sensors on top.

Fig 1 shows the base station for the 12 sensors. The system allows the concurrent use of up to 16 sensors with radio signals transmitted and received over a distance of up to 5 meters. The sensors communicated with each other in 5 second intervals, using a 250 ms window for each sensor to send it’s ID. Transmission of each information package took no more than 100 ms. Shifts of the internal timers of the sensors (< 500 ms per day unused; < 100 ms during measurement) were compensated for by a “base station” that served as a synchronization system and was installed in the medical module EU100 of the isolation chamber. The sensors were synchronized by the base station during the initialization procedure in the morning.

Each WLGS sensor box contained a rechargeable battery (1.5 V, 660 mA), and a standard USB-2 connector for data transfer. The size of the sensor box was 85mm x 46mm x 16mm, and it weighed 70g. There were two different sensor modules. The crew member module could be attached to clothes with a clip at the module’s back. The habitat modules were attached to their designated positions in the isolation chamber with adhesive tape.

All sensor modules worked within a 2.4 MHz frequency band. Modules recorded (a) signal strength (in dB) of the received signals from other transceivers, (b) their identity, and (c) a time-stamp. A 3-axial acceleration sensor registered the orientation of the sensor in space. Changes in orientation were operationalized as activity of the subject. The maximal operating time of the sensor modules in use was 20 hours. Recharging the integrated Lithium-Polymer battery (capacity 660 mAh) took approximately 2 hours at a rate of 400 mA. Data transfer was performed while charging the batteries. A wired network and a host computer (notebook) were used for controlling the system. Participants were provided with three different holders for the sensors: a neck band, a belt, and a clip. However, the participants usually chose to carry their sensors in their pant pockets.

### Mars500 experiments of other PI’s used for comparison

In another Mars500 experiment, Basner et al. [56, 57] continuously registered actigraphy. However, the internal system time of the WLGS-system and the ActiGraph-System shifted differently over the whole 520-days study phase. Therefore the comparison of the internal actigraphy data of the WLGS system with external actigraphy data required a synchronization of both systems data on a daily basis. The maximum of their cross correlation function was corrected for each day separately towards the zero-lag position by adding a day specific time constant to the WLGS data.

For the comparison of the WLGS-data with classical sociometric data (choices) data from the Vinokhodova et al. Mars500 experiment [58] were used. The sociometric questionnaire asked for personal preferences with whom of the crew members to spend the next mission (professional cohesion) and the next vacation (social cohesion). The pairwise registered time spent together was sorted and recoded in analogy to choices. The largest time was recoded as a “2” (respective first choice answer in the questionnaire), the second largest time as “1” (second choice). All other times were recoded as “0”. Correlations were calculated for both sociometric questions over the 30 possible pairings.

For safety reasons the crew was constantly surveyed by several video cameras. For technical reasons the system overwrote these sequences after a few weeks, so at the end only the last weeks were still available. The organizers (IBMP) as well as the crew members gave their consent to use the video data of one whole day (mission day 475) to validate the WLGS data. A late day of the study was sufficient assuming that the WLGS measurements before had the same quality. By means of the INTERACT video analysis software (version 9, Mangold International, Arnstorf, Germany) the WLGS data files were read for comparison with video files. The raw data of day 475 for all sensors were exported separately in one-hour segments. The video films, captured from the Mars500 survey system via a screen capture program (VLC), were edited with regard to the same time intervals and stored as standard video files. Data files and video-files were individually synchronized for each one-hour segment.

Tafforin’s Mars500 experiment [59] provided ethological video analyses of the crew’s inter-individual behavior (whole visual interactions, body interactions, and object interactions) during a collective activity (breakfast) monitored every other week. Analyses were performed using quantitative descriptions with the OBSERVER XT software. This method assessed the number of interactions among crew members and the duration of interactions during the breakfast.

### Data processing and statistical analyses

The primary WLGS data were electronically stored on the sensors’ memory cards and then downloaded to the host’s hard disc. The data were transferred to a server of the Mars500 Experiment Control Center, and then electronically forwarded to the PI on a regular basis. For statistical analyses the data were exported with the application software. Quick graphs (sociogram graphs) were directly provided by the software. Further analyses were mainly performed with IBM SPSS Statistics, version 20 (IBM Corporation, New York, USA). Some analyses and graphs were carried out in the “R” statistical environment (version 2.9.2, www.r-project.org). Latent space models with non-binary response were calculated with the ergmm procedure of the R-package latentnet. This approach is based on Bayesian inference statistics. The main principle is to assume an a-priori distribution (the “prior”, usually a binomial distribution), to combine this with the observed likelihood of measures and to simulate a large enough sample of possible events–the posterior distribution. Different algorithms exist for this simulation; however, for this study the Markov Chain Monte Carlo algorithm was used. To simplify, the posterior is proportional to the likelihood times the prior. The procedure ergmm simulates a posterior distribution of ‘latent positions’ of the crew members. Based on this simulation data statistical comparison of the two natural subgroups by nationality and location of the study (Russians = hosts, Non-Russians = guests [15]) could be calculated and tested to see whether their relations differed significantly.

Data were read and merged per day in separate files. The measured signal amplitudes of other sensors were recoded into “1” (presence) and “0” (absence). The result was a data matrix for each sensor containing presence and absence of other sensors in a five second interval (sample rate = 0.2 Hz). The sum of the “presence” values (multiplied by 5 seconds) provided the measure of the time spent together. In parallel the movements of the sensors were registered by an internal three-dimensional accelerometer.

WLGS data, ethological data of Tafforin’s Mars500 experiment and sociometric data [58] were sampled twice a week, twice a month and monthly respectively. Resampling of the three time series data was required to yield equidistant measurement points, which then were subjected to correlation analysis. Oversampling was used to provide equal statistical power for the time series. The necessary splining approach used the original data points as fixed ones.

All validation data were synchronized per official mission day. Data are presented as means and their standard deviation with α set to 0.05.

## Results

**Fig 2:**
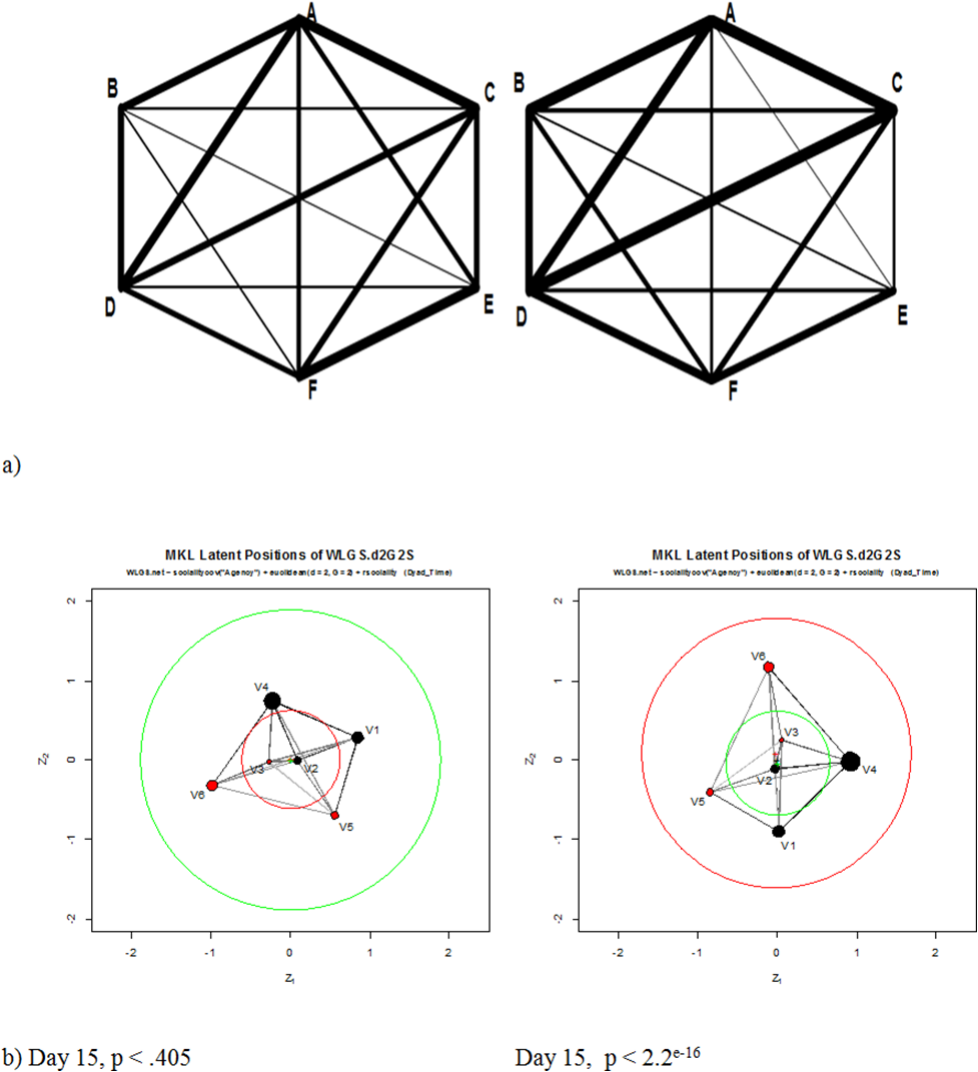
Graphical representation of WLGS data. a: Graphical representation of the time spent together based on data from the six crew sensors on mission days 197 (upper left), and 204 (upper right). These graphs are similar to Moreno’s classical sociogram. The thickness of lines represents the time spent together separately for each measurement day. b: Two graphical representations of subjects latent position and interaction size for one and the same mission day (15) using R statnet including a statistical comparison of two agency groups (“hosts”, “guests”) with extremely opposite significance estimations. For anonymity new subject identifier were randomly assigned (V1-V6).

**Fig 3:**
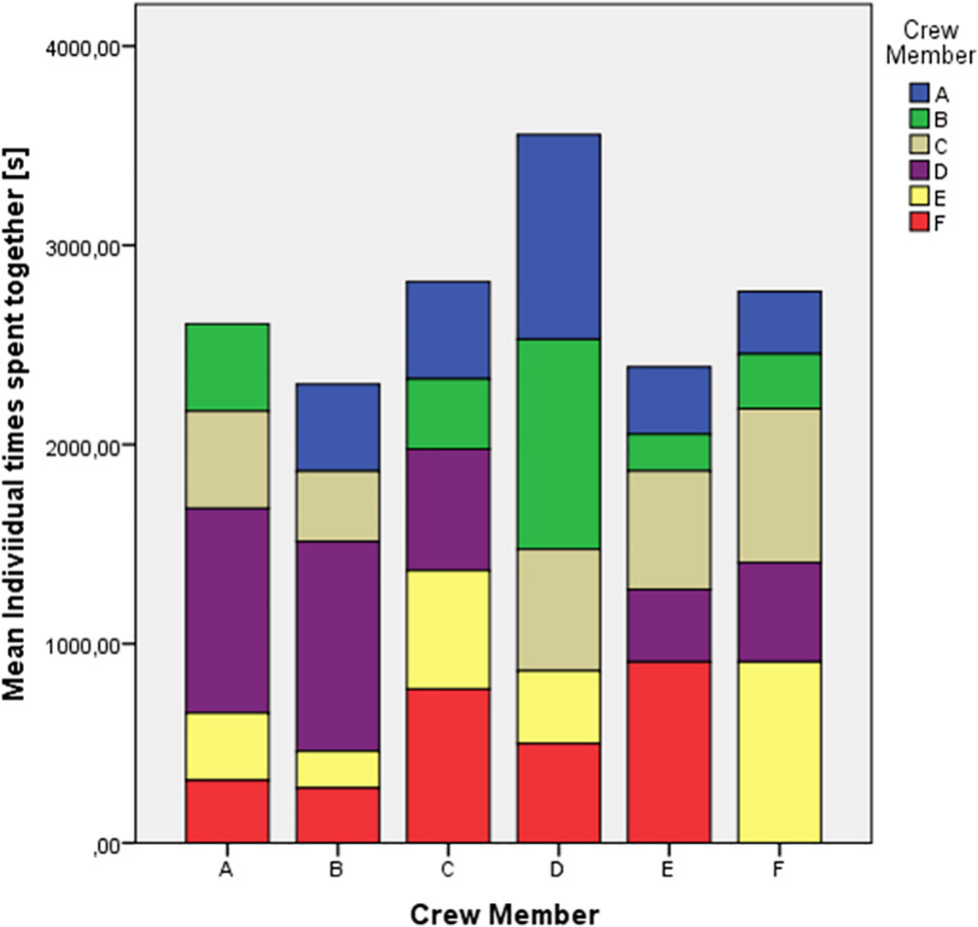
Mean time spent together. Mean time spent together with other crew members during the whole study.

WLGS data were obtained twice a week starting with mission day 15. Out of 132 expected data sets 130 (98.5%) were obtained. However, only 89 (67.4%) were complete during day time for the analysis. The remaining 42 data sets were missing primarily due to insufficient battery recharging. The WLGS data matrix can be found in the S1 Data. Graphs similar to Moreno’s sociogram were generated based on the WLGS data including the crew members only. This kind of classical sociogram illustrates immediately the change of time relationships among the crewmembers. Fig 2A shows some examples of single day assessment. It can be seen that some of the relationships (e.g. A-D, C-D) remain relatively dominant while others (e.g. D-E, B-C) remain relatively weak. In Fig 2B the latent positions of crewmembers were calculated twice for one and the same day (day 15) using the ergmm procedure of the R-package latentnet (statnet). The reference distribution was chosen as recommended by Krivitsky in a short e-mail communication. All crew members were assigned to an “agency”, the “hosts” or the “guests”. The structures look similar but the estimate of the covariate coefficient of the agency effect provided opposite significance results for the F-value (MCMC sample size = 4000): highly significant (p < 2.2e^-16^) vs. not significant (p = .405). The lower Bayesian Information Criterion (BIC) 187.8 vs. 210.7 supports the model with the high significant differences. The averaged time spent together with other crew members is more formally illustrated in Fig 3.

**Fig 4:**
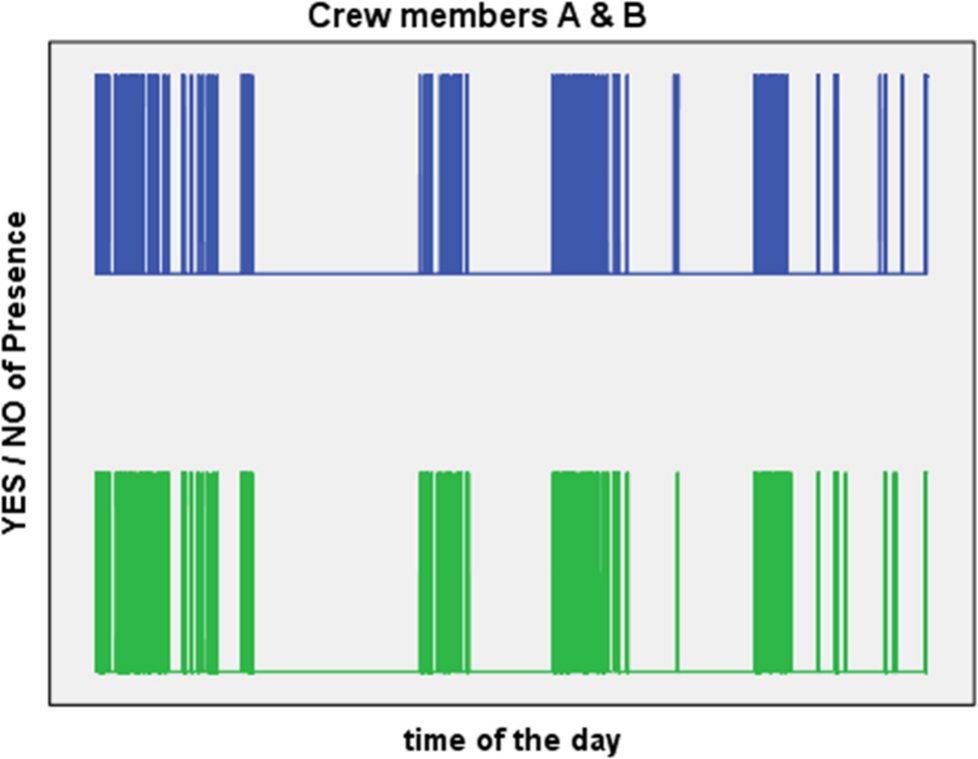
Comparison of two sensor’s data. Presence–absence detections between two sensors (crewmembers A and B) over one mission day registrations.

For internal verification the amplitude data were recoded using the binary system (0 vs. 1). The binary data series of all sensor pairs were correlated with each other. The maximum correlation observed was 0.98 and the averaged correlation of all possible pairs was 0.72 and found to be significant (p = .0056, Pearson's rho). Congruence was defined as the percentage of mutual registration between two sensors, normalized by the total count of registrations. The average congruence was 91.0%. Fig 4 illustrates the 0–1 data of two sensors.

In an exploratory analysis, the reliability of the WLGS activity measure was compared with actigraphy data that was collected by the crew 24/7 every day of the mission [56, 57]). The systems of WLGS and ActiWatch used the different system times of their host computers. The different system times and their time shifts over 520 days were compensated by means of cross-correlation.

In external reliability verification for mission day 475, the crew localization was verified by video and compared to the registrations of the habitat sensors. Eleven hours of WLGS data recording from ca. 9:30–20:30 were analyzed. Table 1 shows the percentage of time that a crew member was registered by the respective habitat sensor if he appeared on the survey video. Private locations such as bedrooms or restrooms were not filmed and excluded from this analysis. The overall percentage of congruent localization was about 95%.

**Table 1:** Congruence of localization between video recordings and WLGS data.

Time interval	Percentage of correct detections	Number of Correct Detections	Number of Failed Detections
**09:18–10:18**	100.0	31	0
**10:18–11:18**	100.0	44	0
**11:18–12:18**	100.0	2	0
**12:18–13:18**	92.3	13	1
**13:18–14:18**	100.0	16	0
**14:18–15:18**	97.2	36	1
**15:18–15:18**	93.9	33	2
**16:18–17:18**	100.0	27	0
**17:18–18:18**	88.9	18	2
**18:18–19:18**	66.7	3	1
**19:18–20:18**	89.5	38	4
**Summary**	96.0	261	11

Percentage and counts of correct and failed detections of location by the WLGS-system are given for mission day 475. The late date during the study was chosen assuming that all measurement before had at least the same detection quotes.

For sociometric analyses the interaction of subjects is most relevant. However, social interaction was relatively sparse before 2 p.m. Technically, WLGS assessment started after the breakfast, and the registrations, as confirmed by the parallel video analyses, were more correct before noon, but this was strictly related to the work schedule. The group’s social life, by contrast, started only in the afternoon and increased until beyond the end of the WLGS registrations, as indicated by the survey video. Thus, as morning activity and interaction was mainly dictated by the work schedule, it would not be very meaningful for WLGS validation to use correlation analysis. Therefore, we limited the sociometric validation analyses of the WLGS data to the afternoon period (14:18 to 20:18), as presented below.

The sociometric questionnaire [58] data were compared with three sets of WLGS data available prior and after the day of questionnaires administration. The correlations increased when WLGS data were limited to periods of actigraphy that indicated crewmembers were “ACTIVE” and not sleeping or resting. This discrimination was impossible based on the internal actigraphy of the WLGS sensors. Table 2 presents the comparison between the monthly questionnaires and the three WLGS measurement days before and after the questionnaire was filled out by the crew members. Significant correlations were found, however, both strength and dependency on the time lag relative to the day on which the questionnaires were filled out varied substantially with time in mission. In all correlations 24.3% (37 of 152) were significant and 7.2% (11 of 152) were on tendency level. For 6.5% of all WLGS measures the correlations were significant with social as well as task related choices of the questionnaire. This amount of significances is highly above the likelihood of occasion (5%) in multiple comparisons.

**Table 2:** Correlations between sociometric questionnaire and WLGS.

Sociogram	r / sig	-3 MD	-2 MD	-1 MD	+1 MD	+2 MD	+3 MD
**1**	Next mission	.333 / .072	.055 / .774	.411 / .024	.236 / .210	.110 / .564	.000 / 1.00
	Holidays	-.063 / .742	.012 / .950	-.144 / .446	.072 / .703	.105 / .581	.188 / .319
**2**	Next mission	.173 / .359	.046 / .811	.000 / 1.00	-.125 / .510	-.146 / .442	-.274 / .143
	Holidays	.152 / .423	.556 / .001	.456 / .011	.091 / .631	.237 / .207	.376 / .041
**3**	Next mission	.128 / .501	-.046 / .811	.046 / .811	.548 / .002	-.046 / .811	.227 / .227
	Holidays	.447 / .013	.365 / .047	.320 / .085	.493 / .006	.137 / .471	.117 / .538
**4**	Next mission	-.265 / .157	.133 / .484	.523 / .003	.073 / .701	-.100 / .598	.219 / .245
	Holidays	-.118 / .535	.277 / .138	.286 / .125	.451 / .012	.271 / .148	.216 / .252
**5**	Next mission	.620 / .000	.396 / .030	-146 / .442	.375 / .041	.292 / .118	.667 / .000
	Holidays	-.003 / .986	.593 / .001	.420 / .021	.283 / .130	.219 / .245	.338 / .068
**7**	Next mission	.110 / .564	.375 / .041	.042 / .827	.146 / .442	.125 / .510	-.042 / .827
	Holidays	.376 / .041	.228 / .225	.173 / .359	.456 / .011	.329 / .076	.100 / .598
**8**	Next mission	.208 / .270	.256 / .173	.210 / .096	.091 / .631	.292 / .117	.402 / .028
	Holidays	.241 / .200	.133 / .482	.086 / .650	.553 / .002	.153 / .419	-.137 / .469
**10**	Next mission	.083 / .662	.230 / .222	-.021 / .913	.292 / .117	-146 / .442	.042 / .827
	Holidays	.091 / .631	.177 / .349	.310 / .095	-.144 / .448	-.037 / .848	.155 / .413
**12**	Next mission	-.219 / .245	.500 / .005	.064 / .737	.456 / .011	.313 / .093	.383 / .036
	Holidays	-.004 / .983	.128 / .501	.096 / .614	.152 / .423	.119 / .532	-.044 / .812
**13**	Next mission	.104 / .584	.042 / .827	.375 / .041	.042 / .827	-.042 / .827	.236 / .210
	Holidays	.228 / .225	.155 / .413	.602 / .000	.402 / .028	.429 / .018	.108 / .569
**14**	Next mission	.149 / .431	.176 / .354	.676 / .000	.521 / .003	.091 / .631	.246 / .189
	Holidays	.095 / .619	.491 / .006	.332 / .073	.009 / .962	.096 / .614	.156 / .410
**15**	Next mission	.210 / .265	.602 / .000	.271 / .148	.226 / .229	.110 / .564	-.105 / .582
	Holidays	.572 / .001	.252 / .179	.206 / .274	-.198 / .293	.152 / .423	.186 / .324
**16**	Next mission	-.072 / .705	.125 / .510	-.042 / .827	.064 / .737	.083 / .662	.064 / .737
	Holidays	.152 / .422	.420 / .021	.320 / .085	.308 / .098	.475 / .008	.248 / .186
**17**	Next mission	.021 / .913	.018 / .924	.354 / .055	n.a.		
	Holidays	.511 / .004	.192 / .309	.146 / .441			

Spearman correlations and significances (r / sig) between the classical sociometric questionnaire of Vinokhodova et al. (2013) and WLGS data; MD = measurement days of WLGS prior or after the day of questionnaire application. The questionnaire was never applied exactly at a WLGS-day. The WLGS MDs were within two weeks before and after the questionnaire respectively.

For a comparison of the CRTI with results of the latent position analysis five series of ergmm models were calculated for 49 randomly selected mission days. The intercept value of the ergmm models was taken as analogue for the CRTI. Significant correlations were found among the ergmm time series, but there was no correlation with the CRTI scores.

**Fig 5:**
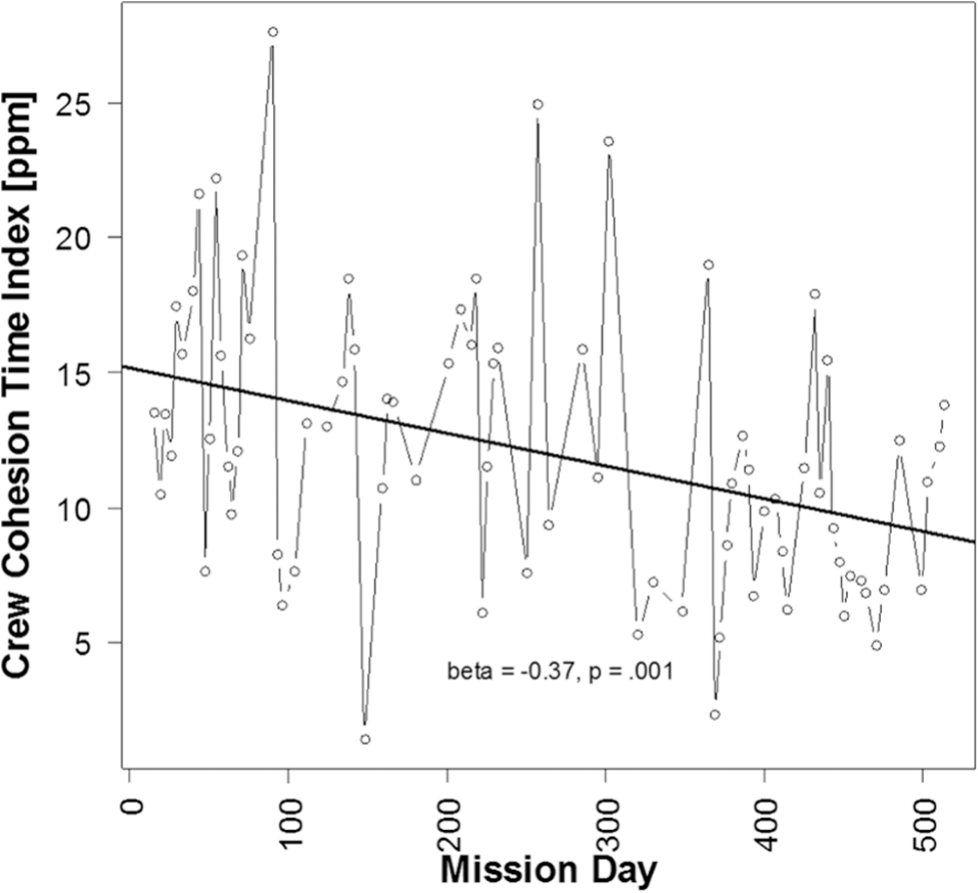
Numeric representation of WLGS data. Numeric representation as integrated Crew Relation Time Index (CRTI) in [parts per million of measurement time] for each measurement day. The central decrease (slope = -0.376) was significant (p = .001).

The WLGS data were also validated by comparing integral cohesion measures over the study period. The integrated index for crew relations (the Crew Relation Time Index, CRTI), based on the WLGS measurements across Mars-520 is depicted in Fig 5. As can be seen from the figure, CRTI fluctuated considerably, and moderately decreased throughout the study (regression over all analyzed mission days: slope Beta = -0.376, p = .001, R^2^ = .141).

The CRTI data were compared with ethological analyses of videos during breakfast. Tafforin [59]) assessed the number of interactions among crew members and the duration of interactions during breakfast. However, the timing of the different methods (WLGS and breakfast video analysis) differed between the methods and was not equidistant. Therefore, the oversampling and resampling method was used to create time series with congruent values for each day.

Significant correlations of day-by-day resampled indexes of crew cohesion in WLGS (CRTI) and ethological breakfast video analysis [59]) were found. The correlation between CRTI and the amount of crew interactions during breakfast was .320 (p = .000). Also a correlation of .230 (p = .000) was found between CRTI and the duration of these crew interactions during the breakfast. The correlation between resampled CRTI data and sociogram data did not reach statistical significance.

Additionally, Table 3 presents the individual correlations over the study period between subjects’ interaction duration with other crew members during breakfast and the time they spent together with other crew members during that day in the WLGS measurement period.

**Table 3:** Individual correlations between the ethological method and WLGS measure.

	A	B	C	D	E	F
**r**	**.113**	-.042	**.165**	**.155**	-.005	.023
**P**	**.011**	.350	**.001**	**.001**	.914	.602

Individual correlations between over-splined and re-sampled data of interaction duration during breakfast (ethological method) and time spent with other crew members (WLGS measure) over mission duration; in three of six participants the individual correlation was low but statistically significant.

**Fig 6:**
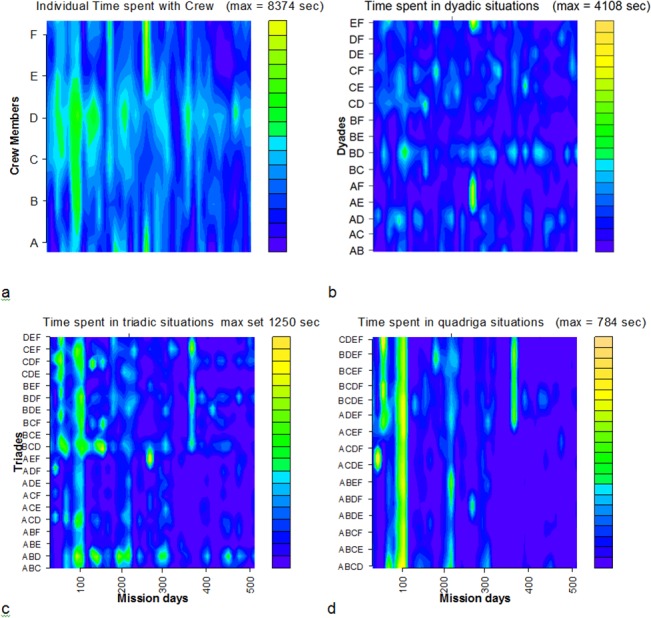
Group relation changes. Graphical representation of whole group relation changes over the study period at one glance: (a) individual time spent with any other crew member; times of interactions in (b) dyadic, (c) triadic or (d) quadric subgroups. The maximum of triadic and quadric times were single events and the automated scale did not provide well-structured graphs. Therefore the graphic scale maximum was set at 1250 sec and 784 sec, respectively.

**Fig 7:**
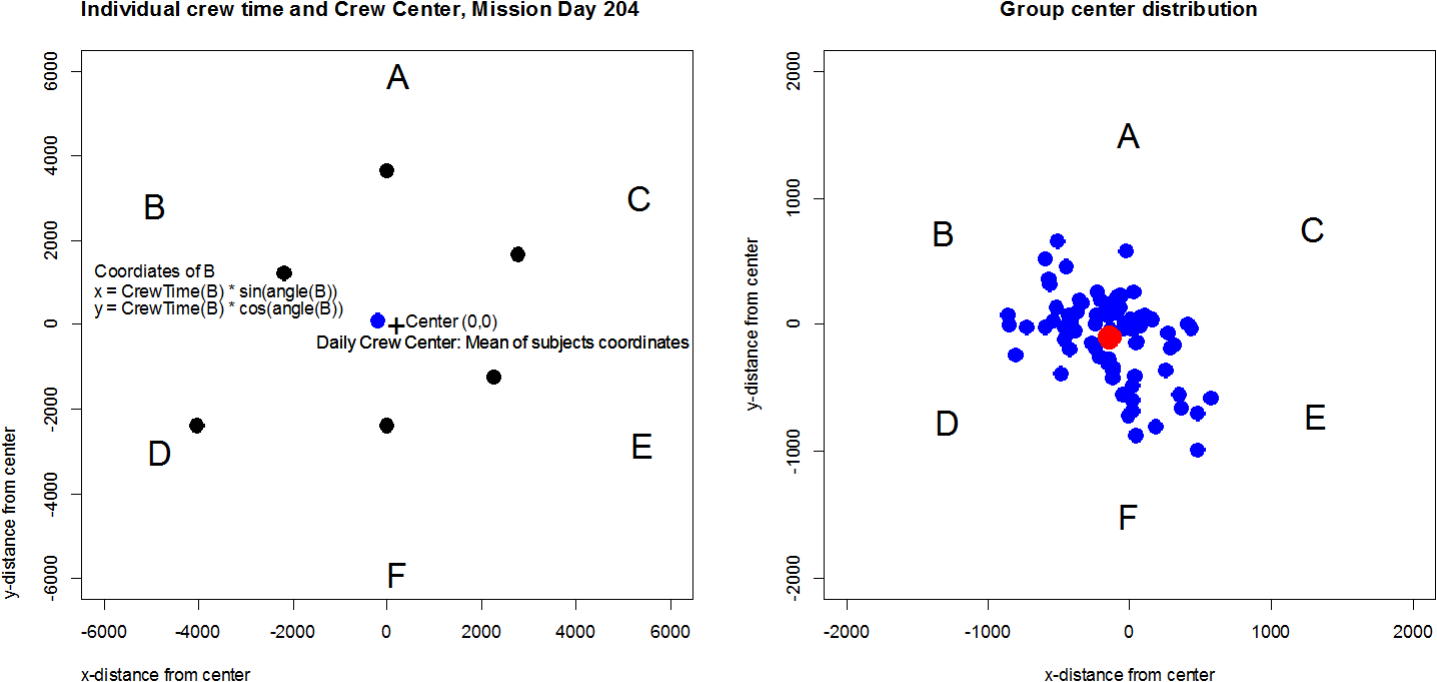
Group relation averages. Left: Calculation of subjects position in the star plot; black dots at mission day = 204: x = crew time (subject) * sin(star-angle(subject)), y = crew time (subject) * cos(star-angle(subject)); daily center of the group (blue dot): mean of coordinates of all subjects; right: daily crew centers of all measurement days (blue dots) and the overall group center (red dot) among crew members during the whole study period.

These correlations were low but statistically significant in three out of six individuals. In a further analysis step visualization methods were adapted which are based on the proportional scaling level of the obtained time data (Figs 6 and 7). Similar graphs were used by Yamamoto & Yokoyama [11].

Fig 6 shows a visualization of the changes of crew structure for intuitive visual monitoring, allowing one to follow changes in time spent together by the crew members. The figure demonstrates that the main group interactions were dyadic interactions. Some dyadic pairs occurred repeatedly over the study period, but were never constant. Triadic and quadric interactions occurred much less frequently (note that the scales for them are strongly zoomed), and showed a clear dominance in the first half of the study and changed dynamically. This supports the ethological results on preferential relationships between crew members [60]). With regard to group structure, a static approach to determine the group center and its changes over the study period was developed. As illustrated in Fig 7, the group center was found to be relatively well centered over the whole study period but also showed daily variance.

**Fig 8:**
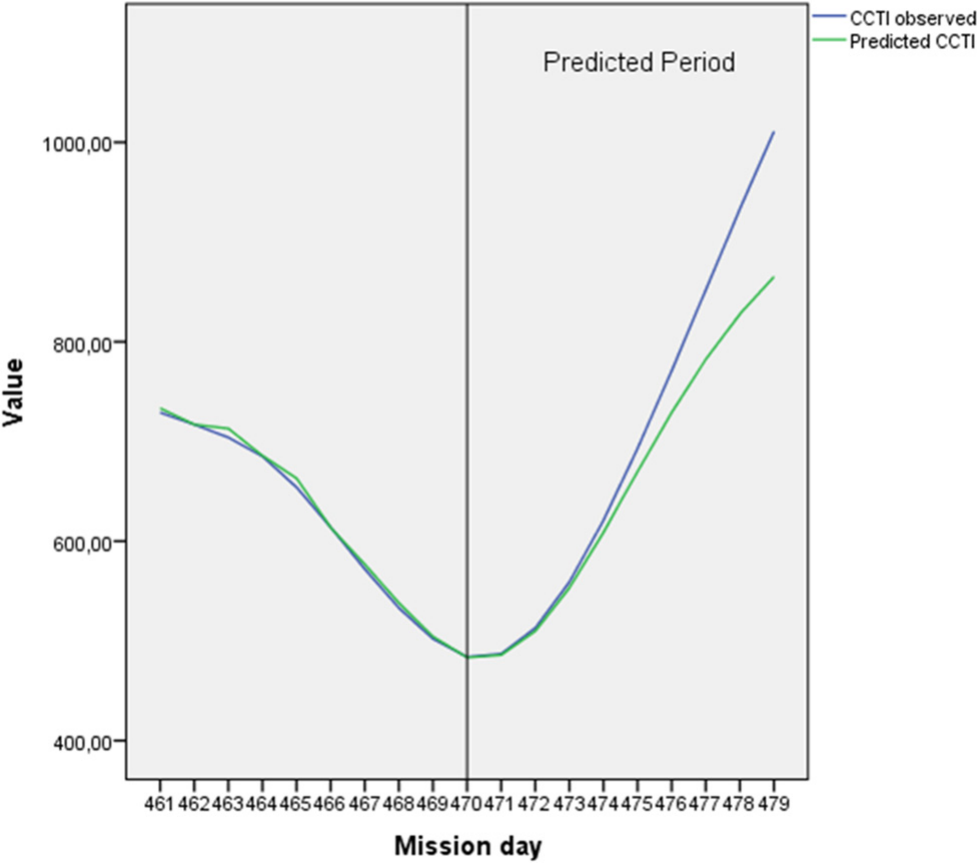
Crew relations forecasting based on WLGS data. The ARIMA model for forecasting of CRTI was based on data from mission day 15 to 470 (shown here beginning with day 461; for whole time series see S1 File). On day 470 the last deep minimum of CRTI occurs. The forecasting was verified with observed data of the predicted period mission day 470–515. For the predicted period predicted and observed values could be found in good accordance of for the next 3 to 4 days.

Fig 8 presents results of an ARIMA model forecasting the CRTI (additional details see S1 File). In the common figure of CRTI mission day 470 was found to be the deepest minimum for more than 100 days (see Fig 5 or S1 Fig C for whole study duration). One could assume a further decrease and a breakdown of crew cohesion; therefore this cut point was chosen. However, ARIMA could predict that the CRTI would increase again afterwards. For the next 3 to 4 days, a good estimation of the observed CRTI values could be given.

## Discussion

The very first application of the HealthLab-compatible nonobtrusive assessment of interpersonal relationships of small autonomic crews during the Mars500 project provides initial evidence for its reliability and validity. This is a contribution towards autonomous and automated crew support systems. Based on the higher scaling level of the WLGS data, possibilities for a visual and numeric monitoring of group structure changes during long-term missions are also presented, promising practical benefits.

The use of analysis methods based on exponential random graphic models was not found to be as useful as a monitoring tool in the present state. As illustrated in Fig 2B, the two graphical representations of the same day (calculated twice with the same approach) do not look similar. The statistical effect of differences between the two subgroups (“agency”) was estimated counter-intuitively. This result is possibly due to the small group size or an improperly chosen reference distribution. However, this analysis of valued ties among the vertices is similar to a Bayesian inference approach and promises new possibilities for statistical group structure evaluation. Still, this must be further developed by statisticians and is beyond the scope of this paper.

The feasibility of the radio-sensor based measurement system was demonstrated, and the reliability of the measurements, including the actigraphy measures, was tested by internal and observational verification. Fig 5 shows a decrease in CRTI over time which is consistent with Basners’ et al. report concerning crew hypokinesis in the same study [56].

Overall, the results support the idea that the time measures were obtained in a sufficiently reliable way for practical application although improvements are desired to increase the measurement interval over 24 hours. The hypothesis about the validity of the “time spent together” measurement as an indicator for personal relationship intensity was initially confirmed by comparison with other sociological measures. Due to the lower scaling level of the sociometric data the daily WLGS data therefore had to be reduced in quantity and scaling level from an interval-scaled measure to an ordinal one. However, the results still demonstrated significant relationships. During the 105-day pilot study [61–62], five of six possible correlations within one-day between WLGS data and the classical sociometric data [58] were significant. No such correlations were found during the 520-day period when comparing sociometric results directly with unselected WLGS data. Significant correlations appeared only when limiting WLGS analysis to time periods in the afternoon and relating them to the “ACTIVE” state of actigraphy. The main reason for the lack of correlation in the whole-day data is thought to be due to the large portion of “private time” during the morning hours with a remaining stochastic variability in dependency on the work schedule. The agreement between WLGS registrations and the video analysis, instead, was highest during the morning phase. The participants stayed longer in a certain location for their work. During the spare time there was much more quick movement within the chamber. Subjects, e.g., were crossing the kitchen only (visible in the video) but the sample rate of the WLGS was too low for an assessment. Table 2 illustrates that from six WLGS measurement points around a day of sociometric assessment there are always some correlation with the sociometric choices. This is to be an important hint that working-time and spare-time have to be evaluated separately (task cohesion vs. social cohesion, [20]).

For a correlative validation of the WLGS data with sociometric (questionnaire) and ethological (breakfast video) data over the time of the study, transformation of their time series were required. Whereas single day comparisons between CRTI and sociogram data provided significant correlations the correlation between resampled CRTI data and sociogram data did not reach statistical significance. The changes in the sociogram data had a too long periodicity, if any. The ethological data showed higher periodicity and significant correlations with the WLGS data.

Validating the WLGS approach in comparison to other measures of cohesion was difficult. In view of the very small sample size, it was unreasonable to expect highly consistent results across individuals and measures. The study design was far from ideal for validation analyses as none of the correlates were synchronized with the WLGS data during the preparation phase and data sharing started only after the study. Unfortunately, there was a noticeable lack of any objective performance measures or team-based tasks allowing the crew to behaviorally demonstrate cohesion. The fact that any significant relationships were found is noteworthy, but even the statistically significant correlations still account for a modest proportion of variance.

A constructed index assumed to indicate crew cohesion (CRTI) based on individual time relationships provided new analytical possibilities. A sociometric questionnaire needs to be applied in longer time intervals. Video analyses are still very time-consuming. The advantage of a measure which can be obtained nearly continuously without disturbing the crew behavior, allows the application of several additional analyses and prediction methods as illustrated with Figs 6, 7, and 8. Whereas the simple regression of the CRTI over the whole mission time describes a common tendency of crew cohesion to decrease, the regression function is not useful for near future predictions. A prediction of crew cohesion changes, demonstrated in Fig 8, could become very important in future long-duration missions to enhance the chance for successful in-time interventions and countermeasures. Predicting time series statistics would enable detection of severe deviations from a stabilized tendency in any of the group features. However, future research is needed to verify this assumption. Alternative methods based on Bayesian inference have to be tested too. In the end, a monitoring and feedback system onboard a spacecraft should focus on the principles of support systems for the flying crew, not as an information system for the ground [48].

The acceptance of such an objective, nonobtrusive measurement method by the crew members is assumed to be much better than subjective reflection requiring answers to questionnaires. It would be desirable to improve the application form of the sensors to enhance the “put-and-forget”-effect.

Finally, the analysis also underlines the necessity to include knowledge about situational and behavioral conditions into consideration–herein task time and spare time. Also, further improvement of the equipment is desired. The registration should ideally require less technical support. The activity diagnostic of the participants should be more detailed as demonstrated by the comparison with external actigraphy. The used sensor is a part of an integrated mobile psychophysiological monitoring system, including voice analysis. This is assumed to be able to enhance the research possibilities in the field of group psychology.

## Supporting Information

S1 Data M520_WLGS_CRTI.csv. (CSV)

S1 File Fig A. Detrended time series CRTI-det of splined CRTI. Fig B. Time series CRTI differences d1_CRTI of splined CRTI, lag = 1. Fig C. Observed and predicted values of the CRTI over the whole study. Fig D. Residual autocorrelation function (ACF) and partial autocorrelation function (PACF) of the stationary l(1) time series d1_CRTI. Fig E. Residuals of the ARIMA model over the whole study. (DOCX)
