# Supplementary material for: Wireless Monitoring of Changes in Crew Relations during Long-Duration Mission Simulation
Source: PLoS One. 2015 Aug 7;10(8):e0134814. doi: 10.1371/journal.pone.0134814 (PMC4529101; doi:10.1371/journal.pone.0134814)
Supplement: S1 File — (DOCX) [file pone.0134814.s002.docx]

**S1 File. WLGS ARIMA forecasting**

The first step of an ARIMA analysis is to determine the stationarity of the time series. The significant decrease of the CRTI (paper figure 5) rejects the stationarity for the original data. The complete time series had a length of 500 days (from day 15 to day 514). The time series was split at day 257 into two halves. The independent samples Mann-Whitney U Test rejects the null hypothesis that the distribution is the same in both halves (p< .001).

One possibility to construct stationarity is detrending. Using the regression parameter of the splined data of CRTI (slope differs slightly from the slope of the original data in figure 5) a new time series CRTI_det (CRTI-detrended) was calculated.

CRTI_det = CCT_S *-0.299354. (S1)


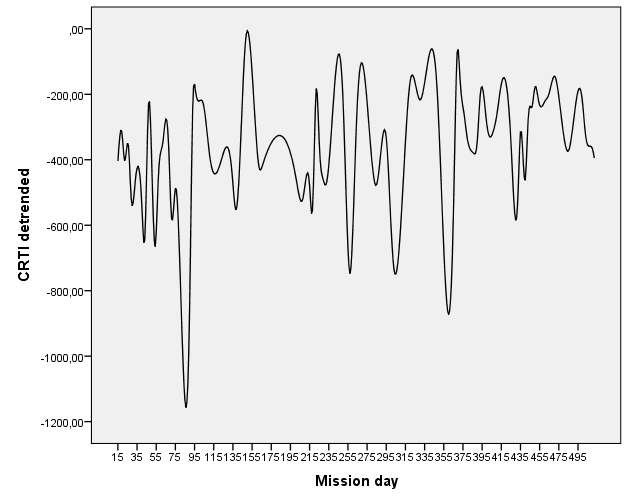


*Figure A. Detrended time series CRTI-det of splined CRTI*

However, the independent samples Mann-Whitney U Test still rejects the null hypothesis that the distribution is the same in both halves (p< .001). Another way to construct stationary time series is the so called differencing, calculating the differences between original data values with a lag of 1 or more. This is the main approach used by the ARIMA method (I stands for the integration of these differences)

d1_CRTI = CRTI – lag(CRTI) (S2)


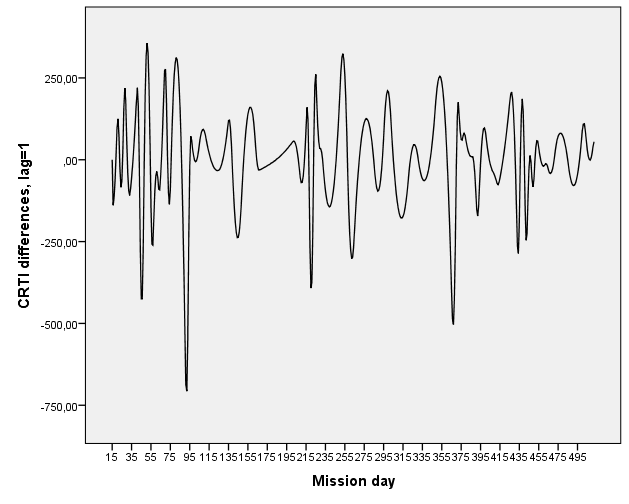


*Figure B. Time series CRTI differences d1_CRTI of splined CRTI, lag = 1*

Now, the independent samples Mann-Whitney U Test confirmed the null hypothesis that the distribution is the same in both halves (p = .996).

By means of the Augmented Dickey-Fuller (ADF) Test it is possible to verify stationarity directly for the whole time series. The ADF-test is not available in SPSS. Therefore data were exported into csv-format for its use in R. The ADF-test was calculated in R, using the package tseries. It confirmed stationarity of the calculated time series d1_CRTI (Dickey-Fuller = -18.6822, lag order = 1, p = .99, alternative hypothesis: explosive).

Assuming the stationarity of the time series d1_CRTI one can identify the initial model of the ARIMA. The ARIMA(p,d,q) combines autoregression (AR, p lags) and moving averaging (MA, q lags) over integrable (I, d steps) time series of differences. The initial model ARIMA(5,1,4) was identified using the whole time series (500 days). We will present here only results if the model’s application the shortened time series until day 470. The day was selected because it represented the deepest minimum for more than 100 days and one could expect a further decrease and a possible collapse of the crew cohesion indication danger for the mission success.

A time series CRTI Test was calculated, setting values for the days 471 till 505 to system missing. The forecasting of this time series for the days 471 till 505 could then be used for the evaluation of the forecasting exactness.

The ARIMA model was confirmed for the CRTI_Test time series with a stationary R-squared about .986 and a Ljung-Box Q value of 9.17 (p = .421) as adequately representing the time series until day 470. Figure S3 illustrates that all ACF and PACF values remained insignificantly with in the confidence interval until a lag of 24.


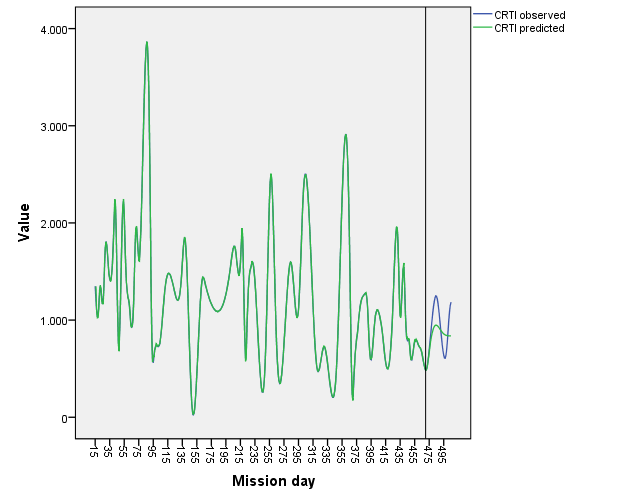


*Figure C. Observed and predicted values of the CRTI over the whole study.*


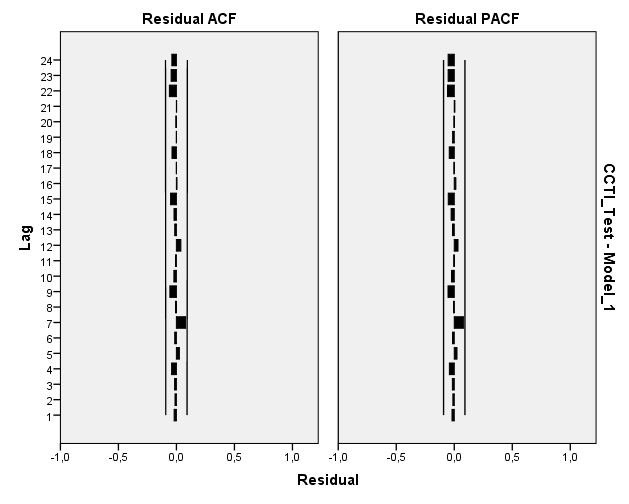


*Figure D. Residual autocorrelation function (ACF) and partial autocorrelation function (PACF) of the stationary l(1) time series d1_CRTI*

A further test for the fit of the model is the stationarity analysis of the residuals (Dickey-Fuller = -15.4112, lag order = 1, p = .99, alternative hypothesis: explosive).

The residuals are normally distributed (Kolmogorov-Smirnov Z = .594, p = .872). One can accept the prediction model as valid.


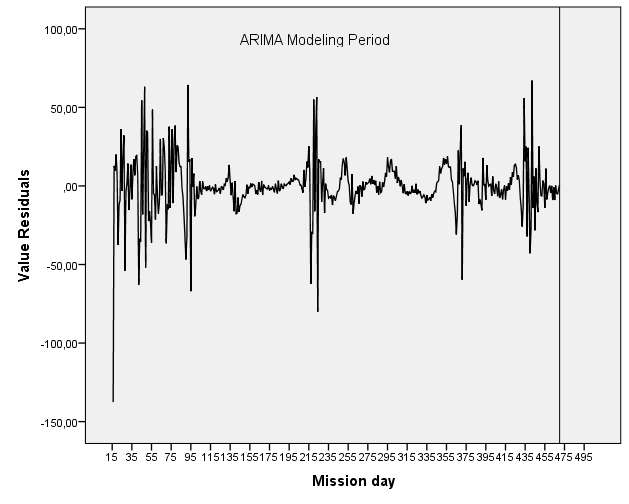


*Figure E. Residuals of the ARIMA model over the whole study*

There could be applied other methods for the evaluation of the fit (e.g. over-fitting). However, for the demonstration of a possible forecasting one can accept the ARIMA(5,1,4) model to be a sufficient fit of the original data (here the d1-CRTI time series) for an evaluation of the forecast values, given in figure 8 of the paper.
